# Preclinical Development of an *In Vivo* BCG Challenge Model for Testing Candidate TB Vaccine Efficacy

**DOI:** 10.1371/journal.pone.0019840

**Published:** 2011-05-24

**Authors:** Angela M. Minassian, Edward O. Ronan, Hazel Poyntz, Adrian V. S. Hill, Helen McShane

**Affiliations:** The Jenner Institute, University of Oxford, Oxford, United Kingdom; Tulane University, United States of America

## Abstract

There is an urgent need for an immunological correlate of protection against tuberculosis (TB) with which to evaluate candidate TB vaccines in clinical trials. Development of a human challenge model of *Mycobacterium tuberculosis* (*M.tb*) could facilitate the detection of such correlate(s). Here we propose a novel *in vivo* Bacille Calmette-Guérin (BCG) challenge model using BCG immunization as a surrogate for *M.tb* infection. Culture and quantitative PCR methods have been developed to quantify BCG in the skin, using the mouse ear as a surrogate for human skin. Candidate TB vaccines have been evaluated for their ability to protect against a BCG skin challenge, using this model, and the results indicate that protection against a BCG skin challenge is predictive of BCG vaccine efficacy against aerosol *M.tb* challenge. Translation of these findings to a human BCG challenge model could enable more rapid assessment and down selection of candidate TB vaccines and ultimately the identification of an immune correlate of protection.

## Introduction

The ethical barriers to challenging humans with virulent replicating mycobacteria have so far negated the development of a human challenge model of *M.tb* infection. This is in contrast to infectious diseases such as malaria, influenza and typhoid, where vaccine developers benefit from the ability to experimentally infect volunteers to assess candidate vaccine efficacy in small scale proof-of-concept Phase II challenge studies prior to expensive field studies [1], [2], [3], [4]. Consequently, the TB vaccine field has to rely on preclinical animal challenge models of *M.tb* infection or on the development of *in vitro* models of *M.tb* killing as surrogate measures of vaccine efficacy [5]. However, it remains uncertain how predictive these are (if at all) of human vaccine efficacy, and the development of a relevant *in vivo* challenge model is urgently required. Such a model has real potential to facilitate TB vaccine development. Here we demonstrate a novel *in vivo* BCG challenge model using BCG vaccination as a surrogate for *M.tb* infection. This is based on the hypothesis that an effective vaccine against *M.tb* should also reduce the replication of BCG. Published studies support this hypothesis: vaccine suppression of a BCG challenge is comparable to that of an *M.tb* challenge, and studies have most commonly assessed the protective effect of the BCG vaccine itself on a subsequent BCG challenge [6], [7], [8], [9]. Importantly, BCG is also a feasible challenge agent for human use: it is a safe replicating mycobacterium (with 99.95% sequence homology to live *M. bovis)* [10]. It causes a self-contained limited infection in immunocompetent animals and humans, and is licensed for human use as an intradermal (*id*) administered vaccine.

While there are clear genetic differences between BCG and *M.tb*, the cell-mediated immune responses elicited by both mycobacteria are very similar. Both organisms are intracellular pathogens controlled predominantly by CD4^+^ T cells, at least in the early stages of infection. Macrophages infected with both organisms attract CD4^+^ and CD8^+^ T cells by MHC class II [11] and MHC class I presentation respectively, and transfer of mycobacterial antigens to dendritic cells (DCs) leads to NK cell activation. IL-17 production from gamma-delta (γδ) T cells and other non-CD4/8^+^ cells is also common to both pathogens, and has been shown to contribute to granuloma formation in lung tissues in the mouse model [12], [13]. Both pathogens have also been shown to induce Tregs *in vivo* [14].

The protection against pulmonary TB afforded by BCG is both variable and incomplete; however, BCG does confer worthwhile protection against disseminated forms of TB [11]. BCG-based regimes, designed to improve the efficacy of BCG either by follow-on with a subunit vaccine boost, or by over-expression of genes within a recombinant BCG, are currently in the forefront of vaccine development. We have tested largely BCG-based regimens (BCG +/− subunit boost) using this challenge model, although application of the model to testing of single dose subunit vaccines and other non-BCG based regimens is feasible (providing the candidate vaccine incorporates an antigen that is also present within BCG).

There are few published data on BCG replication in murine skin, and none beyond two weeks after immunization [15]. Here we have assessed for the first time the replication kinetics over a 12 week period of a murine BCG challenge administered into the skin, using the mouse ear as a surrogate for human skin. We have demonstrated the kinetics of BCG replication with three different doses of BCG and characterized the associated cellular immune response to BCG. The aim was to determine the time of the peak BCG mycobacterial load and use this time-point in subsequent murine and human studies assessing the protective effect of candidate TB vaccines on a BCG skin challenge. By identifying the peak time-point for organ harvest and quantitation of the BCG mycobacterial load, the ability to detect a significant reduction in this BCG skin load would be maximised. A proof of concept in the mouse model was thus established to examine the protective effect of BCG and candidate TB prime-boost vaccination strategies against a subsequent BCG skin challenge.

Our results demonstrate that live BCG persists in murine skin for at least 4 weeks and that *id* BCG immunization consistently protects against a BCG skin challenge, an effect that is independent of immunization dose, route or immunization-challenge interval. Where BCG-based immunization regimens are shown to protect, the reduction in BCG CFU counts in the skin correlates with the pre-challenge CD4^+^ T cell responses to purified protein derivative (PPD) and antigen 85A (Ag85A) in the blood. Moreover, efficacy of BCG immunization against subsequent *id* BCG challenge is predictive of vaccine efficacy against aerosol *M.tb* challenge, suggesting this model may be useful in predicting vaccine effects against *M.tb*.

## Results

### BCG replicates for 4 weeks in the ear and up to 12 weeks in the local draining LNs

**Figure 1:**
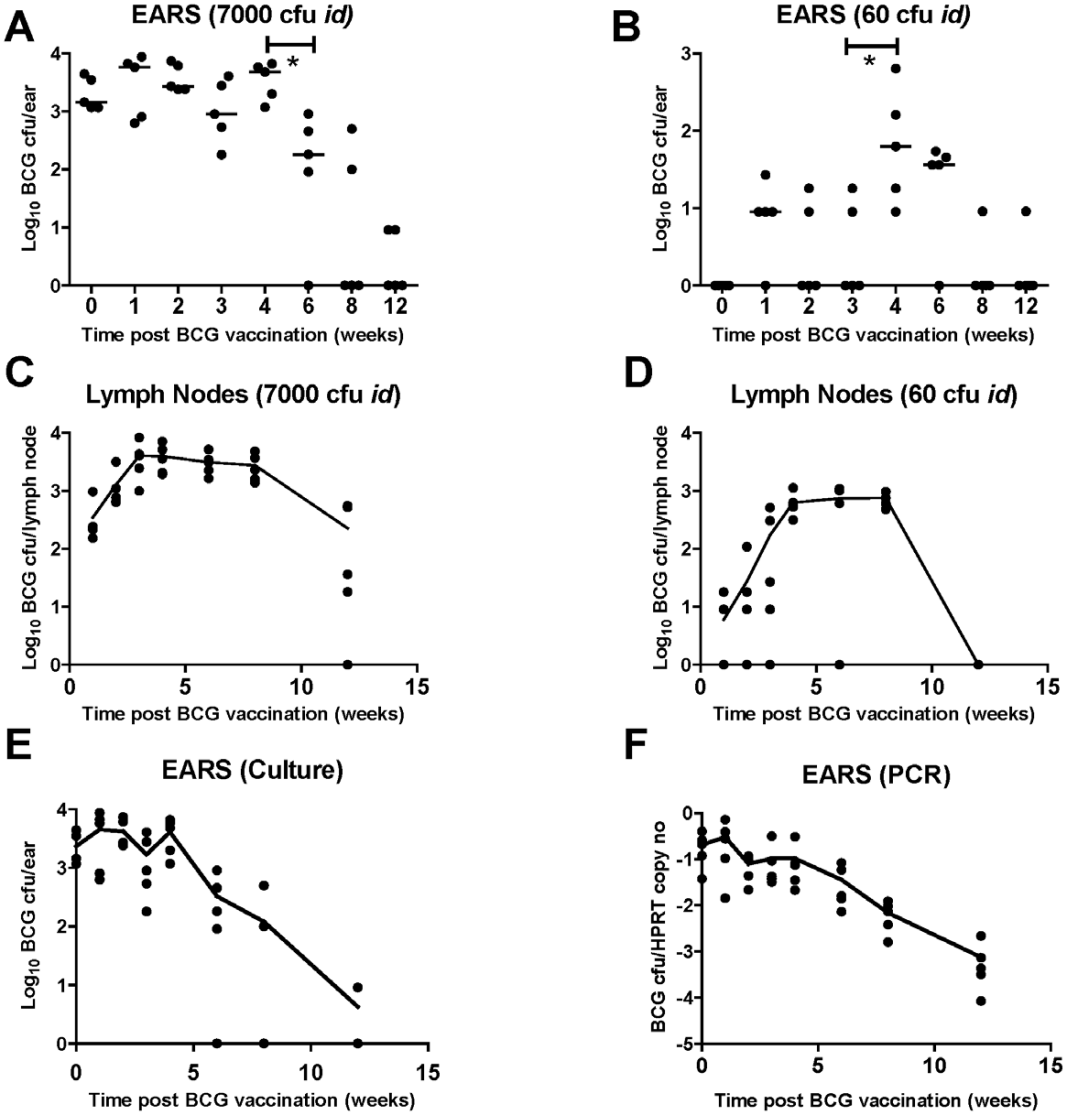
Timescale showing BCG persistence in the ears and LNs of *id*-injected mice up to 12 weeks post immunization. Log BCG CFU in the ears (estimated by culture) are shown for (**a**) the high dose group (7000 CFU *id*); and (**b**) the low dose group (60 CFU *id*). Log BCG CFU in the auricular LNs are shown in (**c**) the high dose group (7000 CFU *id*); and (**d**) the mid dose group (60 CFU *id*). Datasets include individual data points for each mouse; the bars represent the median per group in (**a**) and (**b**), and a line connects the means for each group in (**c**) and (**d**). * indicates *P*<0.05. Both ears and LNs were homogenized and plated onto 7H11 Middlebrook agar. Log BCG CFU in the ears estimated by culture (**e**) and BCG genome copies/mHPRT copies estimated by PCR (**f**) are shown in a timescale for the high dose group, up to 12 weeks post BCG immunization. Quantitative PCR was performed with BCG-specific primers. Individual data points are shown for each mouse with a line connecting the means for each group.

A time-course experiment, measuring out to 12 weeks post vaccination, described the replication kinetics of BCG in the ears, local draining (auricular) LNs and spleen following *id* BCG immunization. Three different doses of BCG were used: 7000, 60 and 1 CFU. The culture data for the high dose (7000 CFU) and mid dose (60 CFU) groups are shown (Fig. 1a and 1b). Live BCG CFU persisted for 4 weeks, after which there was a significant decline in CFU counts (*P* = 0.04). Animals vaccinated with the mid dose (60 CFU) showed significantly diminished levels of BCG throughout the time-course, the levels falling to almost zero after week 4. No CFU were detected at any time-point in animals vaccinated with just 1 CFU of BCG (data not shown). The PCR data are shown for ear CFU in the high dose group (Fig. 1f) and show a comparable kinetic to culture (1e), with a significant reduction in CFU between day 0 and week 6 (*P* = 0.03) and again between weeks 6 and 8 (*P* = 0.009). There was a strong positive correlation between the levels of BCG estimated by the two quantification methods across both high and mid doses (*R* = 0.77, *P*<0.0001, Spearman).

In contrast to replication in the skin, replication in the LNs was greater in the mid dose group (Fig. 1d) and followed a similar kinetic to the high dose group (Fig. 1c). In the high dose group, peak replication was reached by week 3 and maintained until 8 weeks. There was then a significant decline between week 8 and 12 (*P* = 0.009). In the mid dose group, the peak was delayed (6 weeks), but was also maintained until 8 weeks, significantly dropping thereafter (*P* = 0.005).

In summary, live BCG persists in the skin for at least 4 weeks and in the LNs for up to 12 weeks. These results provided a 4-week detection window within which candidate vaccines could be assessed for their ability to protect against a BCG skin challenge.

### Immunogenicity of BCG in the spleen is dose-dependent

**Figure 2:**
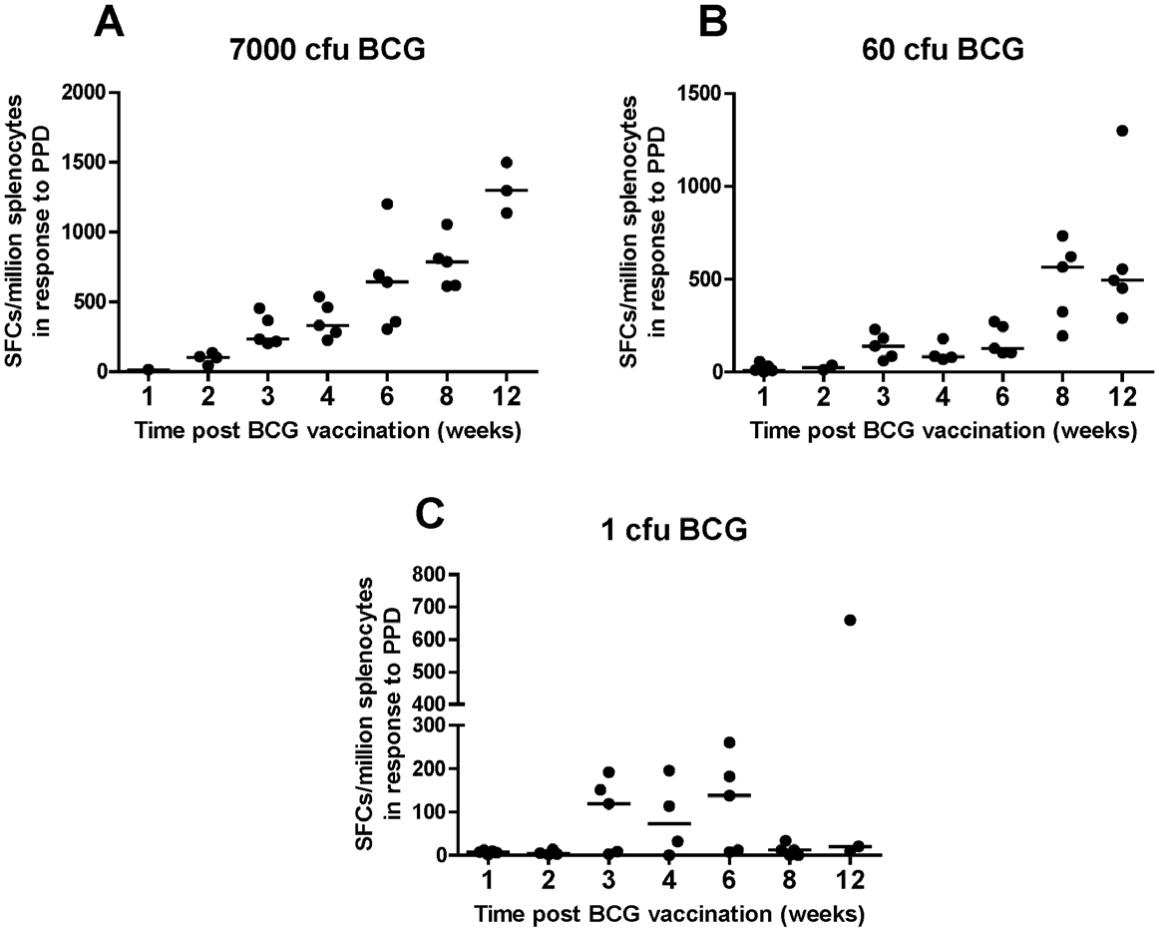
Splenic IFN-γ responses to PPD up to 12 weeks post BCG immunization. In (**a**) high dose group (7000 CFU *id*); (**b**) mid dose group (60 CFU *id*) and (**c**) low dose group (1 CFU *id*). Datasets include individual data points for each mouse; the bars represent the median value per group. Results expressed as SFC/million splenocytes.

There are few published data on the immunogenicity of *id*-administered BCG in the mouse model, and fewer assessing the effect of vaccine dose on immunogenicity. The interferon-gamma (IFN-γ) ELISpot responses to PPD over time in the three groups of animals described are shown in Fig. 2. The high (7000 CFU) and mid (60 CFU) dose groups showed an increase in immune response over time, although the kinetic was delayed in the mid dose group. By week 8, similar levels of IFN-γ responses were observed in both groups (median of 566 spot-forming cells (sfc) per million splenocytes (mid) vs 788 sfc (high)). In the high dose group, the ELISpot responses then continued to increase at the latest (week 12) time-point (median 1298 sfc), whereas in the mid dose group, the responses reached a plateau (median 496 sfc). Even the low dose group (receiving just 1 CFU of BCG) showed a detectable PPD response up to the week 6 time-point, although the response was not maintained beyond this (Fig. 2c). However, one animal demonstrated a persistent IFN-γ response out to week 12. ELISpot responses to the immunodominant BCG antigen TB10.3 mirrored the responses to PPD (data not shown).

### Single dose subunits, MVA85A and Ad85A, fail to protect against an *id* BCG challenge, but BCG immunization protects against subsequent BCG challenge

Having defined a 4-week window for detection of live BCG in the skin, we next assessed the ability of candidate vaccines to protect against an *id* BCG challenge.

**Figure 3:**
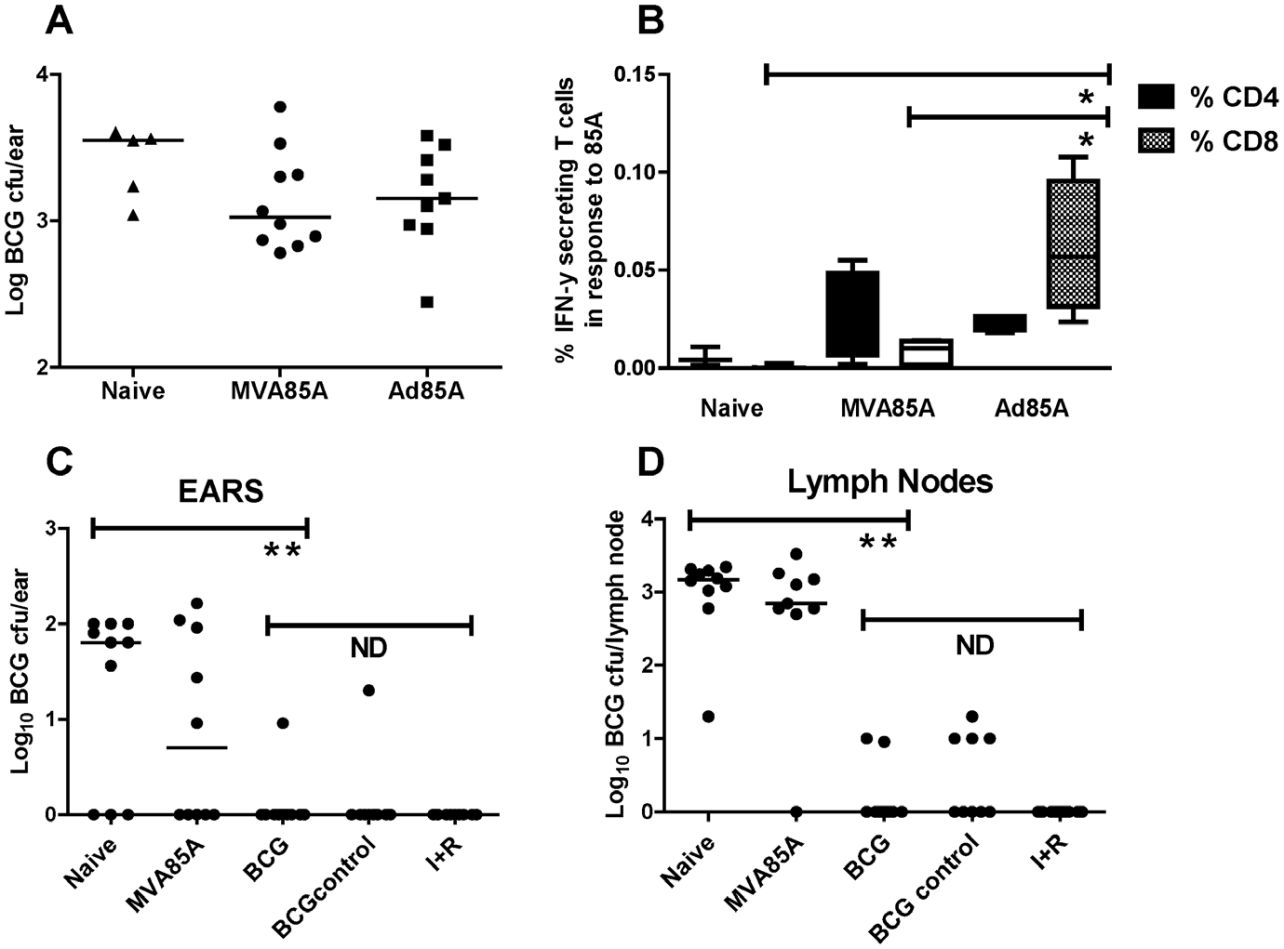
Effect of single dose vaccines (subunits MVA85A and Ad85A, and BCG) on an *id* BCG challenge. (**a**) BALB/c mice were immunized *id* with 1×10^6^ pfu MVA85A or 2×10^9^ vp Ad85A. Control mice (Naïve) received no immunization. Four weeks later all mice were challenged *id* with 1×10^5^ CFU BCG, contralaterally to the site of vaccination. Ears and LNs were harvested 4 weeks after BCG challenge and processed for CFU quantification. (*P<0.05, *n* = 10 except naïves, *n* = 5). (**b**) Corresponding intracellular cytokine staining (ICS) of the local draining LNs. Red bars represent the proportion of IFN-γ-secreting CD4^+^ T cells in response to 85A, blue bars represent the same for CD8^+^ T cells (M, *n* = 4; Naïve, *n* = 3; Ad, *n* = 4. *P<0.05). (**c**) Effect of BCG vaccine compared to subunit MVA85A on an *id* BCG challenge. BALB/c mice were immunized *id* with either 1×10^6^ pfu MVA85A or 2.2×10^4^ cfu BCG. “Naïve” and antibiotic-treated (I+R) mice received no immunization. Four weeks later all mice were challenged with 6×10^3^ CFU BCG, except the BCG control group who received no challenge. In the I+R group, challenge was followed by 4 weeks treatment with isoniazid and rifampicin. Ears and LNs were harvested 4 weeks after BCG challenge and processed for CFU quantification. Log_10_ BCG CFU individual data points for each mouse are shown. Bars represent the median per group. (**c**) Ears (**P<0.01, “non-significant, ND”, *n* = 10); (**d**) LNs (**P<0.01, “non-significant, ND”, *n* = 10).

Mice were immunized *id* with a viral vectored candidate TB vaccine (MVA85A-Modified vaccina virus Ankara expressing mycobacterial antigen 85A [16]-or Ad85A-recombinant E1/E3-deleted adenovirus human serotype 5, AdHu5, expressing antigen 85A [17]), and then challenged four weeks later with BCG. The amount of BCG in the ears of mice four weeks after BCG challenge was then quantified (Fig. 3a). There was a trend for a reduction in CFU counts between naïve animals and those vaccinated with MVA85A and Ad85A, but this did not reach statistical significance (*P* = 0.11 and *P* = 0.16, respectively). Intracellular cytokine staining (ICS) of the local draining LNs showed that the CD8^+^ T cell response to antigen 85A (% of IFN-γ-secreting CD8^+^ T cells) was higher for the Ad85A group than MVA85A or naïve groups (Ad85A vs naïve, *P* = 0.02; Ad85A vs MVA85A, *P* = 0.035). The 85A-specific CD4^+^ T cell responses were similar across both MVA85A/Ad85A groups (Fig. 3b). There was a strong positive correlation between the lymph node ICS and spleen ELISpot responses to 85A (*R* = 0.86, *P* = 0.01, Spearman, data not shown). However, there was no correlation between these LN/spleen *ex-vivo* responses and BCG CFU measured in the ear post challenge (data not shown).

The effect of BCG immunization on subsequent *id* BCG challenge was subsequently assessed in a separate experiment (Fig 3 C&D). A significant reduction in BCG CFU was seen in the group with prior BCG immunization (Fig. 3c) (1.8 logs, *P* = 0.006). A similar reduction in CFU was also seen in the local draining LNs (3.2 logs, *P* = 0.0002) (Fig. 3d). Unimmunized control mice, challenged with BCG and then treated with isoniazid and rifampicin (I+R) for four weeks, had a comparable level of BCG CFU to the BCG vaccinated mice. MVA85A, again administered as a single dose regimen, conferred a non-significant reduction in BCG CFU, in agreement with the previous experiment (Fig. 3a). Residual CFU from the first BCG immunization, shown as the “BCG control” group, did not significantly affect the final CFU result as these CFU counts were negligible (median CFU<10).

### BCG protection against a BCG challenge is dose-independent

**Figure 4:**
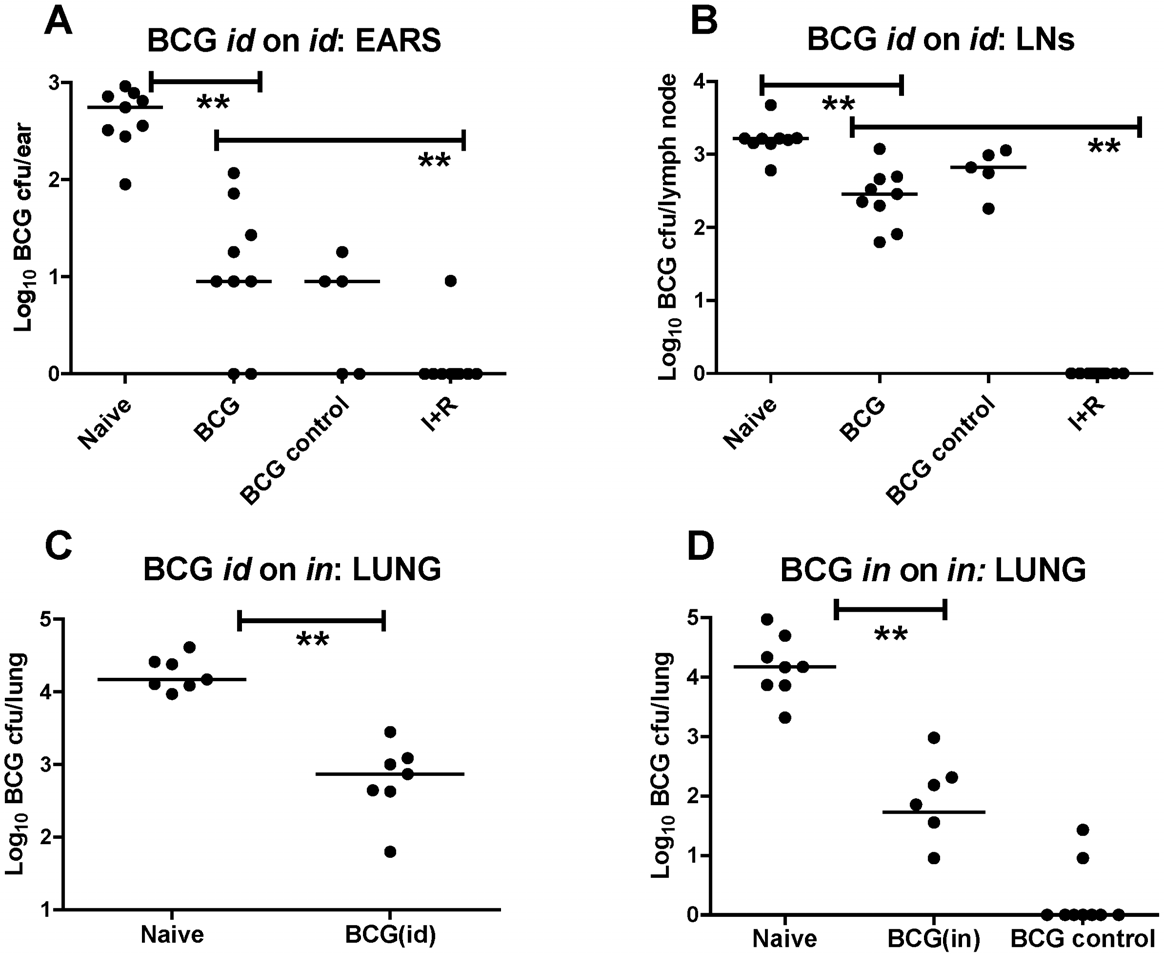
Effect of BCG on a 4-week *id* and *in* BCG challenge. BALB/c mice were immunized *id* with 2.5×10^3^ CFU BCG. Naïve and antibiotic-treated (I+R) mice received no immunization. Four weeks later all mice were challenged either *id* or *in* with 4×10^3^ CFU BCG, except the BCG control group who received no challenge. Immediately post-challenge the I+R group was treated for 4 weeks with isoniazid and rifampicin. Ears (**a**), LNs (**b**) and lungs (**c**) were harvested 4 weeks after challenge (spleen cfu data not shown). (**d**) shows the effect of *in* BCG on *in* challenge in a separate experiment. Here, BALB/c mice were immunized *in* with 1×10^3^ CFU BCG. Naïve mice received no immunization. 4 weeks later *in*-immunized and naïve mice were challenged with 4×10^4^ CFU BCG *in*. The “BCG *in* control” group received no challenge. Lungs were harvested 4 weeks after BCG challenge in all groups. CFU from plating of fresh tissues are shown. Log_10_ BCG CFU individual data points for each mouse are shown. Bars represent the median per group. (**a**) Ears (**P<0.01, *n* = 10); (**b**) LNs (**P<0.01, *n* = 10); (**c**) Lungs (**P<0.01, *n* = 10); (**d**) Lungs *P*<0.01, *n* = 8 naïve; n = 6 BCG *in; n* = 8 BCG *in* control).

We next investigated whether the protective effect of BCG immunization was dose dependent. A comparable reduction in the CFU count of challenge was seen when a 1-log reduced BCG immunization dose of 2.5×10^3^ CFU was administered (*P* = 0.0005, Fig. 4a). The challenge dose administered was the same as for the previous experiment. There was also a significant reduction in the LN CFU counts (*P* = 0.0005, Fig. 4b).

### BCG immunization protects against BCG challenge at a distant site

In the model being developed, an *id* BCG challenge is being used as a surrogate for aerosol *M.tb* challenge. As route of challenge may be important, we next compared the efficacy of *id* BCG vaccination on both *id* and *in* BCG challenge in parallel (Fig. 4c). Prior *id* BCG immunization resulted in a significant reduction in lung CFU (1.3 logs) after an *in* challenge (*P* = 0.002), which was comparable to, though of lesser magnitude than the reduction seen in the skin after an *id* challenge (1.8 logs, Fig. 4a). When the BCG immunization was administered *in* (Fig. 4d), a 2.4-log reduction in BCG CFU in the lung was observed. This suggests that a greater reduction in CFU by BCG may be achieved when immunization and challenge are administered to the same site.

### Effect of BCG immunization on subsequent BCG challenge is durable

**Figure 5:**
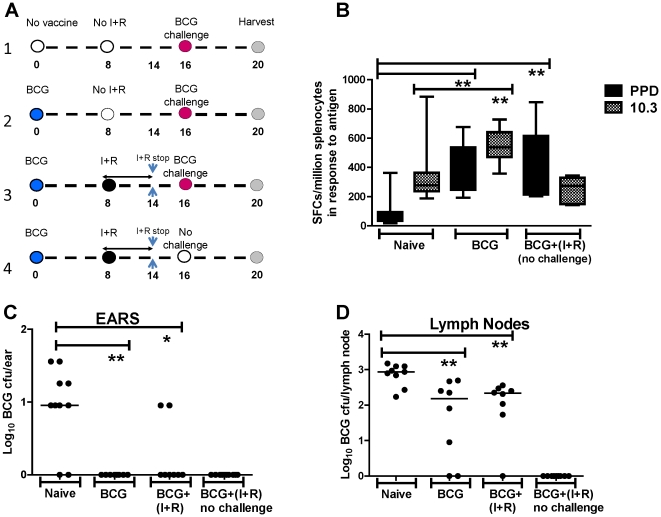
Effect of BCG immunization on a 16-week *id* BCG challenge. Timeline is shown in (**a**). BALB/c mice were immunized *id* with 1×10^4^ CFU BCG. Naïve mice received no immunization. 16 weeks later all mice were challenged with 10^3^ CFU BCG, except the (BCG+(I+R)(no challenge)) group. This group and the BCG+(I+R) group received 6 weeks of isoniazid and rifampicin (starting 8 weeks post initial BCG immunization). In the BCG+(I+R) group, there was a 2 week wash-out period between cessation of antibiotics and subsequent BCG challenge. (**b**) shows the splenic IFN-γ ELISpot responses to PPD and TB10.3, in naïve, BCG-vaccinated, and BCG+(I+R)(no challenge) animals, 4 weeks post BCG challenge. Whiskers represent minimum to maximum values, boxes the interquartile range, and the bars the median values for each group (*P<0.05, **P<0.01, “non-significant”, ND. *n* = 10, except for BCG+(I+R); *n* = 2). Ears and LNs were harvested 4 weeks after BCG challenge and processed for CFU quantification. Log_10_ BCG CFU of challenge are shown, for groups 1 (Naïve), 2 (BCG) and 3 (BCG+(I+R)). For group 4 (BCG+(I+R) no challenge), the ear and LN CFU correspond to the CFU remaining from the priming BCG immunization (zero in all animals). Individual data points for each mouse are shown. Bars represent the median per group. (**c**) Ears (*P<0.05,**P<0.01, *n* = 10); (**d**) LNs (**P<0.01, *n* = 10).

We assessed the durability of protective efficacy by evaluating a 16-week window between BCG immunization and subsequent challenge. Mice were immunized *id* with BCG and then challenged with BCG 16 weeks later, with or without an intervening period of antibiotic therapy (Fig. 5a, timeline). There was a significant reduction in CFU (1 log) in the ears of BCG-immunized mice compared to challenge-only naïves (Naïve:BCG, *P* = 0.002; Fig. 5c). A similar reduction in CFU (0.75 log) was seen in the local draining LNs (Naïve:BCG, *P* = 0.004; Fig. 5d).

Fig. 5b shows the post-challenge ELISpot responses of the same animals (20 weeks post initial BCG immunization in the *id* immunized group). Here the BCG-immunized group (BCG, group 2) demonstrated significantly increased post-challenge responses to PPD, compared to the naïve (challenge-only) group 1 (*P* = 0.001).

These results suggest that the protective effect of BCG immunization on BCG challenge is not only dose-independent, but also independent of the immunization-challenge interval (at the intervals measured: 4 and 16 weeks).

### Antibiotic clearance of live replicating BCG does not reduce vaccine efficacy

Two additional groups were included in the previous experiment to assess the effect of antibiotic clearance of the BCG vaccine on a subsequent *id* BCG challenge (groups 3 and 4, see timeline, Fig. 5a). Data from group 3 (BCG+(I+R)+(no challenge)), showed the effect of delayed antibiotic treatment on the immune response from the prior BCG immunization. Immune responses to PPD between this group and group 2 (who received no antibiotics after BCG immunization and also went on to receive a challenge) were comparable. In addition, there were significantly higher PPD responses in these groups compared to the naïve (challenge-only, group 1) animals (*P* = 0.005). Again, efficacy was maintained in the group receiving antibiotics (4): Naïve: BCG, *P* = 0.005, Naïve: BCG+(I+R), *P* = 0.01.

These results demonstrate that the administration of antibiotic therapy 8 weeks post BCG immunization is sufficient to kill all remaining viable bacteria, but does not attenuate the immune-mediated efficacy of the priming BCG immunization.

### Efficacy in the skin produced by BCG and BCG-MVA85A/Ad85A regimes correlates with PPD and Ag85A-specific pre-challenge CD4^+^ T cell responses in the blood

**Figure 6:**
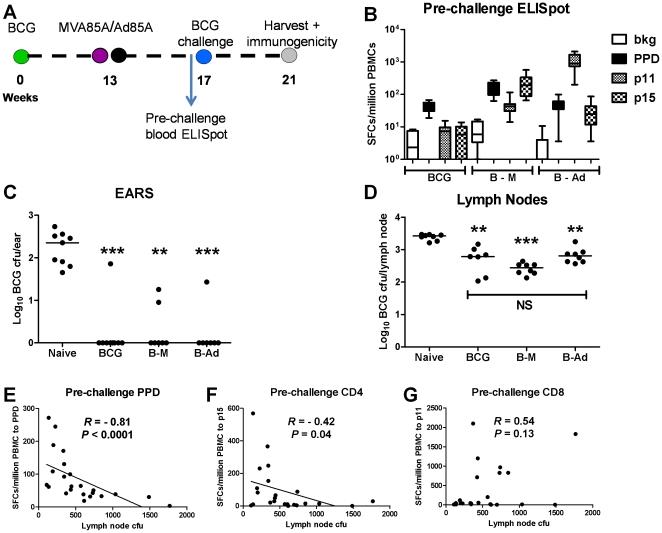
Effect of BCG, BCG-MVA85A (B–M), and BCG-Ad85A (B-Ad) prime-boost regimes on an *id* BCG challenge. Timeline shown in (**a**): BALB/c mice were immunized with 10^4^ CFU BCG *id* and then boosted after 13 weeks with either 1×10^6^ pfu MVA85A *id* or 2×10^9^ vp Ad85A *id*. All animals were challenged 4 weeks later with 6×10^3^ CFU of BCG *id*. Organs were harvested 4 weeks after challenge. Log_10_ BCG CFU of challenge are shown in (**c**) Ears (***P<0.001, **P<0.01, *n* = 8); and (**d**) LNs (***P<0.001, **P<0.01, *n* = 8, “NS” = no significant difference). **/*** indicate significance of immunization regimes over naïve mice. (**b**) **Pre-challenge immunogenicity as measured in the blood by ELISpot.** IFN-γ responses were assessed after stimulation with PPD, a H-2^d^ CD4^+^ T cell epitope, and a H-2^d^ CD8^+^ T cell epitope present in the *M.tb* antigen 85A, on blood samples taken one day pre- BCG challenge. IFN-γ responses within all three vaccinated groups (BCG (**B**), BCG-MVA85A (**B-M**), and BCG-Ad85A, (**B-Ad**), *n* = 10) are shown. Whiskers represent minimum to maximum values, boxes the interquartile range, and bars the median values for each group. Correlations between LN cfu and pre-challenge blood ELISpot responses to (**e**) PPD; (**f**) CD4^+^ epitope; and (**g**) CD8^+^ epitope. Spearman correlation analysis (with individual data-points for all 30 mice in the three vaccination groups) is shown.

The hypothesis that the protective effect of BCG could be boosted by a candidate subunit vaccine was subsequently tested using this challenge model. The effect of prime-boost regimes BCG*id*-MVA85A*id* (B–M) and BCG*id*-Ad85A*id* (B-Ad) on BCG challenge, in comparison to BCG alone (BCG), is shown in Fig. 6. All three regimes achieved comparable reduction in BCG CFU counts in the skin and LNs. (Fig. 6c: Ear CFU: BCG, (*P* = 0.0004), B–M, (*P* = 0.0014) and B-Ad, (*P* = 0.0008); LN CFU: BCG, (P = 0.001), B–M, (P = 0.0008, P = 0.001).

T cell responses were assessed in the blood by ELISpot pre-BCG challenge to allow the potential for identification of a pre-challenge immune correlate. The PPD response to BCG was significantly boosted by MVA85A (*P* = 0.001) but not by Ad85A (*P* = 0.29 Fig. 6b). The response to a H-2^d^ CD4^+^ T cell epitope from antigen 85A induced by BCG was significantly boosted by both MVA85A (*P* = 0.0008) and Ad85A (*P* = 0.006), but was significantly higher in the B–M compared to B-Ad group (*P* = 0.002). Similarly, the response to a H-2^d^ CD8^+^ T cell epitope from antigen 85A was boosted by both subunits; Ad85A (*P = *0.0008), MVA85A (*P* = 0.001), but was significantly higher in the B-Ad compared to B–M group (*P* = 0.0008). This is in agreement with the known predilection of these subunit candidates for a predominantly CD4^+^ (MVA) or CD8^+^ (Ad) biased T cell response. There was a significant negative correlation between the pre-challenge PPD response and the level of challenge BCG CFU in the draining LNs, in immunization groups BCG, B–M and B-Ad (*R* = −0.81, *P*<0.0001, Fig. 6e). There was a weak negative correlation between the pre-challenge CD4 response to the CD4 epitope and challenge CFU (*R*  = −0.42, *P* = 0.04, Fig. 6f). No significant correlation was observed between the pre-challenge response to the CD8 epitope and challenge CFU (*R* = 0.54, *P* = 0.14, Fig. 6g).

In summary, subunit booster vaccines MVA85A and Ad85A failed to significantly augment the protective effect of BCG alone (at the challenge dose assessed). However, efficacy in the skin produced by both BCG alone and BCG-MVA85A/Ad85A regimes correlated with the pre-challenge CD4^+^ T cell responses to PPD and Ag85A.

## Discussion

We have presented a novel *in vivo* challenge model for testing candidate TB vaccines. This model was developed as a preclinical model of a human challenge model, and the data and techniques presented in this manuscript formed the basis of a clinical study which successfully translated this approach into the human setting (Minassian *et al.*, submitted).

We demonstrate the persistence of BCG in the skin for up to four weeks at a level that allows for suppression by a protective vaccine regimen, and our model suggests that protection against a BCG skin challenge (by prior BCG-based immunization) is predictive of vaccine efficacy in the lung. This latter finding supports the development of a mycobacterial skin challenge as a model for *M.tb* exposure and challenge.

Our results show for the first time that live BCG persists in the skin for at least four weeks, and in the lymph nodes for up to 12 weeks, the latter coinciding with the peak splenic T cell immune response. Whether detection of live BCG at the vaccine site can be taken as evidence of true in-situ replication is an important question. The natural course of BCG replication in the spleens of *iv-*vaccinated mice is a 0.5-1-log increase in CFU in the first 10–14 days. However, the peak CFU level reached is comparable to the dose initially administered, with no significant increase beyond this. Similarly, in the lungs of *iv* vaccinated mice, there is a 3-log increase in BCG replication over the first 4 week period, but again the peak CFU level reached is comparable to the dose administered [6]. We found no significant rise in CFU in the mouse ear after day 0 with no true detectable peak, but similar to the previous published work, the numbers of live BCG detectable in the first 4 weeks are not different from the levels seen at day 0. There is likely to be a balance in the level of bacterial replication and clearance during this period-the clearance being attributable to either bacterial death and phagocytic clearance, or phagocytic transport of live bacteria to the local draining LNs. This may in part explain the different kinetics in the LNs, where there is likely to be a greater proportion of live bacteria and so where BCG replicates to much greater levels, compared to the ear where there are relatively few lymphoid cells and where phagocytic clearance may have a prominent role. Recent work has demonstrated the use of an endogenous bacterial enzyme probe in rapid detection, imaging and quantification of *M.tb* and BCG within tissues of a living host. This could potentially play a role in quantifying BCG/*M.tb* challenge over time *in vivo* and ultimately may provide an alternative method for quantifying mycobacteria in mice [18].

This model uses BCG administered *id* as a surrogate for challenging with *M.tb* by aerosol. We therefore evaluated how predictive the protective effects seen in the BCG *id* (skin) challenge model are of those seen in the aerosol (lung) challenge model, by investigating the effect of BCG on both ***id*** and ***in*** BCG challenge in parallel. We have demonstrated that suppression of a BCG skin challenge can be achieved by prior BCG immunization, consistently resulting in a 1–2 log reduction in CFU. This effect is mirrored in the local draining LNs, and is similar to the magnitude of suppression that *id* BCG exerts on a distant lung challenge. This protective effect (of BCG *id* on BCG *id* challenge and of BCG *id* on BCG *in* challenge) also mirrors the effect of parenteral BCG on *M.tb* aerosol challenge in previous published studies [7], [19], [20], [21]. This latter finding suggests that if a BCG-based vaccine regimen is protective against *M.tb*, its effect on suppression of a BCG skin challenge may predict its effect on an aerosol challenge.

Our experiments have shown the protective effect of BCG to be independent of the route or dose of BCG administered, and also of the immunization-challenge interval. Longer interval experiments should be performed to better test the protective effect of BCG-induced central and effector memory T cell responses. Olsen *et al*. demonstrated that immunity measured in the spleen declined in BCG-immunized mice when the microorganisms were cleared by antibiotic therapy, compared to non-antibiotic-treated animals, where immune responses were maintained until 9 months [22]. While we have shown the immunogenicity *not* to be affected by antibiotics (in spite of successful eradication of all live BCG), we have only measured this at one time-point post BCG immunization (20 weeks). There were additional differences between ours and Olsen's experiments: Olsen *et al.* cleared BCG with antibiotics at 15 weeks post-immunization and continued the antibiotics for 8 weeks, whereas in our model antibiotics were started 8 weeks post-immunization and continued for 6 weeks. Our mice were also vaccinated by the *id* as opposed to *sc* route. However, in agreement with Olsen's data, we show that antibiotic clearance of live replicating BCG post-immunization does not ablate efficacy against a subsequent mycobacterial challenge (as measured by BCG challenge CFU in the skin and LNs (our data), and *M.tb* challenge CFU in the lung (Olsen *et al.*)) This suggests that once generated, protective immunity to BCG is maintained, even in the absence of replicating live mycobacteria. It will be important to determine the minimum time window required for immune engagement by BCG and development of protective immunity.

Our prime-boost immunogenicity data indicate a correlation between pre-challenge PPD/Ag85A-specific CD4^+^ T cell responses in the blood and efficacy (reduction in BCG CFU counts) in the skin. However, the phenotype of such “protective” CD4^+^ T cell responses has yet to be fully defined. Multiple gene knockout (KO) studies, cell depletion and adoptive transfer experiments in mice demonstrate the importance of CD4^+^ T cells in protective immunity against TB [23], [24], [25], [26], [27], [28], [29], [30]. However, the precise mechanism of CD4^+^ T cells in protection against mycobacterial challenge remains to be defined.

While the dose of BCG does not affect its protective capacity, we have shown that it does affect the peak organ bacterial load, in agreement with other published studies [31], [32], [33], [34]. Challenge dose is important, as efficacy of any immunization regimen may be affected by dose. This was demonstrated by our prime-boost experiment of BCG-MVA85A and BCG-Ad85A on BCG challenge. Here, improvement over the suppressive effect of BCG alone by these *id*-administered subunits was not achievable at a challenge dose of 10^3^–10^4^ CFU, in these relatively small numbers of animals, as the protective effect of BCG is so profound. This is in agreement with published data on the effect of parenterally-administered BCG-MVA85A/Ad85A compared to BCG alone against *M.tb* challenge in the mouse model [19], [35], [36]. These data therefore support this skin model in its prediction of BCG-based vaccine effects against lung challenge with *M.tb*. A non-significant effect on BCG *id* challenge also mirrored the lack of effect on *M.tb* aerosol challenge (seen in previous studies) for the single dose subunit regimes tested, e.g., single dose parenterally-administered Ad85A [36], [37].

The convincing effect of BCG against BCG challenge in this murine model has been validated against the extensive literature on the effect of BCG against *M.tb* challenge in mice. This finding is very relevant for many mycobacteria-based vaccine regimens currently in development (including recombinant strains of BCG and attenuated strains of *M.tb*).

Given the purpose of this study is to support the development of a *human* challenge model, further assessment of protective non-BCG-based candidate subunit vaccine regimens (which do not replicate /disseminate like BCG and may protect via different mechanisms of action) may be more feasible in humans and more relevant non-human primate pre-clinical models. In these models, the known variable effect of BCG should allow more scope for improvement in protection by a candidate subunit vaccine or BCG booster regimen, compared to the mouse model where the effect of BCG is consistently profound.

The relationship between protective immunity in the skin to that in the lung remains to be fully ascertained. However, comparisons of the effect of *id* BCG vaccination against *id* and *in* BCG challenge in this work support a predictive relationship between efficacy against *id* BCG and aerosol *M.tb.* While comparative parallel studies of the effects of new candidate vaccines against challenge with BCG *id* and *M.tb* aerosol, would validate this *id* model in prediction of effects against aerosol *M.tb* challenge, there is extensive published work by several groups over the last few decades showing that the data against BCG challenge in this paper (with BCG) accords with the effects of BCG against *M.tb* challenge.

In summary, we have assessed the ability of candidate vaccines to protect against a BCG skin challenge in mice, and BCG itself has been shown to consistently protect. This mirrors published data of the effects of BCG on *M.tb* challenge, supporting the relevance of a mycobacterial skin challenge to an aerosol *M.tb* challenge for assessment of BCG-based vaccines. These findings are now being applied to a human model of BCG challenge, where replication of BCG in human skin has been characterized and its replication within an immunized group of volunteers is being assessed (Minassian *et al.*, manuscript submitted). A challenge model such as this which allows vaccine assessment could be enormously valuable in candidate TB vaccine down selection, and better still if an immunological profile associated with reduced BCG bacterial load in the skin could be identified.

## Materials and Methods

### Animals and Immunizations

All procedures were carried out under the terms of the UK Animals (Scientific Procedures) Act Home Office Project Licence (UK Home Office PPL 30/2412) and were approved by the University of Oxford Animal Care and Ethical Review Committee.

The studies described used 6–8 week old female BALB/c (H-2^d^) (Harlan, UK). Animals were vaccinated with one or more of BCG, recombinant MVA85A, or recombinant Ad85A. 25–30 µl of vaccine was inoculated into the ear dorsum. Animals were then challenged with BCG *id* into the contralateral ear. All intranasal exposures were performed in a class I hood. Mice were removed and immunized with 50 µl given drop-wise to the nares.

The doses of each vaccine used, unless otherwise stated, were 10^1^–10^5^ CFU of BCG, 10^6^ plaque-forming units (pfu) recombinant MVA85A or 2×10^9^ viral particles (vp) Adeno(AdHu5)85A [38]. Viable freeze-dried BCG vaccine SSI (Danish strain 1331) was used for all experiments.

### Tissue harvest and homogenization

Mice were sacrificed by cervical dislocation and tissues (lungs, spleens, ears, lymph nodes (LNs)) removed by dissection in a sterile fashion and homogenized. Lungs, LNs and spleens were beaten in a mini-bead beater (Glen Mills inc) for 1 min in 2 ml V-bottom cryovials (Starstedt) containing 1/3 glass beads and 1 ml Dulbecco's phosphate-buffered saline (DPBS). Dilutions of tissue homogenates were then made 10-fold in PBS (900 µl PBS, 100 µl tissue); 1 dilution for spleens and 2 dilutions for ears, LNs and lungs. 100 µl of each neat sample and each dilution were plated onto 7H11 agar containing 10% OADC supplement (E&O laboratories). Plates were incubated at 37°C for 3–4 wk and the number of BCG colonies (CFU) counted.

Ears were homogenized in a dispomix machine (Thistle Scientific) after transfer into dispomix tubes containing 1 ml of sterile PBS. A 2-step program was applied: Program 5– involving a 20 s gradation spin/cut cycle; Program 11– involving 90 s of homogenization (fibrous tissue specific). The homogenate was then sonicated at half power for 15 s.

### DNA extraction of BCG from tissue

200 µl of a 1 ml tissue homogenate (mouse ear or human skin) in DPBS was beaten for 4 min in a mini bead-beater (Glen Mills Inc), until smooth. 180 µl of ATL (tissue lysis) buffer and 20 µl proteinase K (PK, Qiagen) were added, vortexed and the sample incubated at 56°C in a shaking heating block for 4 h. The sample was then heated to 95°C for 15 min to inactivate the PK. Once cooled, chicken egg-white lysozyme (Sigma), (final concentration 0.5 mg/ml), was added and the sample incubated for 1 h at 37°C. After **v**ortexing for 15 s, 400 µl AL buffer & 400 µl 100% ethanol (premixed) were added to the sample and vortexed. From this point, Qiagen manufacturer's instructions were followed. The DNA was eluted with AE buffer (200 µl). After incubation for 1 min at room temperature, the column was centrifuged at 8000 rpm for 1 min. The elution was then repeated with a further 200 µl of AE buffer to maximize DNA yield, making the final sample volume of DNA 400 µl. The sample was frozen immediately at −20°C.

### Primers

**C3/5** primers, designed by Magdalena et al [39], are specific for the senX3-regX3 region, an intergenic region (IR) separating two genes encoding a mycobacterial two-component system. Depending on the BCG substrains examined, BCG Danish has been shown to give a product of 276-base pairs (bp), whereas strains of *M. bovis* and *M.tb* give a larger product of 329+ bp.

**ET 1 and 3** are complementary to regions flanking the BCG deletion RD1 sequence. In strains without RD1 (all strains of BCG) they bind and amplify a 196-bp region, but in strains with RD1, the 9650-bp sequence is too big to efficiently amplify [40].

Using primer design software the published sequences were modified to minimize the risk of self-binding and primer dimer formation. Table 1 shows the final sequences of these BCG-specific primers (showing base pair differences from the original published sequences underlined).

**Table 1:** Final sequences of BCG-specific primers, C3/5 and ET1/3, showing base pair differences from the original published sequences underlined.

	Primer Sequence
C 3/5 (forward)	5′ –GAA CTG C GG TCA AAC AGG TCA CAA C -3′
C3/5 (reverse)	5′ –AGC GAC TCC TCG TCC TCC ACA ATC -3′
ET 1/3 (forward)	5′ –CCG CCG ACC GAC C TG ACG AC -3′
ET 1/3 (reverse)	5′ –GGC GAT CTG GCG GTT TGG GG -3′

### PCR reaction

5 µl of each DNA sample (neat, 1∶10 or 1∶100 dilutions) was added to wells in a 96-well plate, each well containing a mixture of 10 µl of SyBr Green mastermix (Sigma), 3 µl of RNAase-free water (Sigma) and 2 µl of a 10 pmol/ µl mix of both forward and reverse primers (MWG biotech), giving a final reaction volume per well of 20 µl. A 15 min denaturation step (at 95°C) was followed by 50 cycles of amplification, including an annealing step at 60°C for 30 s and an extension step at 72°C for 30 s, followed by melt and cooling programs. To control for variation in DNA quantity between samples the copy number of the gene of interest was divided by the copy number of the house keeping gene mHPRT. All PCR reactions were performed in duplicate.

A standard curve was made by extracting BCG DNA from serial 1 in 10 dilutions of BCG. Five fresh vials (each containing ∼1×10^6^ CFU) were together reconstituted in 1 ml PBS. Serial 1 in 10 dilutions were made from this starting stock (100 µl into 900 µl PBS), from ∼5×10^6^->5×10^−2^/ml. DNA was extracted from 200 µl of each 1 ml dilution, culminating in final volume of DNA of 400 µl. 5 µl of each 400 µl DNA sample was subsequently used for each PCR reaction. Therefore 5 µl of DNA represented a range from ∼1.25×10^4^–1.25×10^−4^ CFU per PCR reaction (5 µl×80 = 400 µl DNA; 400 µl DNA = 200 µl BCG; 200 µl×5 = 1 ml BCG). Both primer sets produced a linear result, confirming the PCR was efficient, from 1.25×10^3^–1.25×10^−2^ CFU. The standard curve was subsequently corrected for live CFU, by solid culture CFU quantification of the same BCG aliquots. There was >0.5 log discrepancy in CFU between the culture results and the estimated number of BCG genome copies by PCR. The standard curve was therefore adjusted, such that 1.25×10^1^ estimated copies corrected to 2.32×10^0^ live copies of BCG. The PCR limits of CFU detection in a 1 ml tissue homogenate therefore ranged from a minimum of ∼0.9 BCG CFU/ml tissue (0.09 CFU from a plated 100 µl aliquot of tissue) to a maximum of ∼9×10^4^/ml tissue. The lower end of detection by PCR reflected a 10X increase in sensitivity over culture. While the maximum concentration of amplifiable BCG was just under 10^5^ CFU, this was considered adequate for detection of BCG in the skin given an average administered dose of 10^3^ to 10^5^ BCG CFU.

### Antibiotic therapy

Antibiotic preparations of rifampicin (R3501, Sigma) and isoniazid (I3377, Sigma), were added to 400 ml drinking water bottles, to make a final concentration of each antibiotic in water of 100 mg/l. The bottles were then administered to the animals' cages in the usual procedure. This dual therapy was administered to antibiotic control groups of mice for 4 weeks to ensure complete eradication of BCG.

### Cell isolations

Cells from freshly harvested spleens and LNs were resuspended in 5 ml M10 (Modified Eagle's Medium alpha-modification (Sigma), supplemented with 10% heat-inactivated Foetal Calf Serum (FCS), 2 mM L-glutamine, 1% penicillin/streptomycin and 50 µM 2-mercaptoethanol (Gibco)).

Approximately 100 µl of blood was collected from tail veins into 200 µL of 1 M EDTA. Erythrocytes were lysed using Puregene RBC Lysis Solution (Flowgen) and peripheral blood mononuclear cells (PBMCs) harvested by centrifugation at 4000 rpm for 4 min. The cell pellet was resuspended in 500 µl M10. Cells were counted using a CASY counter (Schärfe Systems, Germany) and resuspended in complete medium.

### Mouse *ex-vivo* IFN-γ ELISpot assay

The enzyme-linked immunospot (ELISpot) IFN-γ assay was carried out as previously described [41]. Cells from immunized and naïve control mice were resuspended at 5×10^6^ or 1×10^7^ cells/ml (splenocytes) or 8×10^5^ cells/ml (lung cells) in M10. PBMCs were resuspended at variable concentrations and plated with 0.5 and 0.25×10^6^ naïve splenocytes per well. 50 µl cells were plated in duplicate into the coated wells, serial 1 in 2 dilutions were made if necessary. Cells were assayed following 18–20 h of stimulation with Tuberculin PPD (20 µg/ml, SSI), r85A (10 µg/ml, University of Leiden), and 17 overlapping peptides of TB10.3 and TB10.4. Cells from 5–10 individual mice were tested in each group, and each condition was tested in duplicate. All of the peptides used were 15mers overlapping by 10 amino-acids, used at a final concentration of 10 µg/ml in each well. Stimulation of PBMCs was performed with a H-2^d^ CD4^+^ T cell epitope from antigen 85A (sequence: tfltselpgw lqanrhvkpt), and a H-2^d^ CD8^+^ T cell epitope (sequence: ewydqsglsv vmpvggqssf).

Phytohaemagluttinin (PMA, 10 µg/ml) was used in all assays as a positive control and M10 alone as a negative control. Cells were counted using an ELISpot counter (Autoimmun Diagnostika, Germany).

The ELISpot data were analysed by subtracting the mean number of spot-forming cells (sfc) produced from the negative control wells from the mean count from stimulated wells. A well was considered positive if the count was at least twice that in the negative control wells and at least 5 spots more than the negative control wells. Positive control wells were stimulated with PHA and the control failed if there were not more than 200 spots/well. Results are expressed as either median spot forming cells (sfc) per million splenocytes, lung cells or PBMCs, or as median sfc/spleen or lung.

### Cell stimulations and antibody staining for flow cytometric analysis

Cells were adjusted to a concentration of 1–2×10^7^ cells/ml in M10 and 100 ml/well were plated into a round bottom 96-well plate. 50 ml of GolgiPlug (1∶250 dilution) (BD Pharmingen) was added to each well, together with 50 ml/well of antigen 85A pools at 4 µg/ml final concentration. Plates were incubated at 37°C /5%CO_2_ for 5 h and then refrigerated at 4°C.

Cells were transferred to a V-bottom plate and centrifuged for 2 min, 1800 rpm, blocked with 100 ml Fc-γ receptor block (anti-CD16/32) and incubated for 15 min on ice. Cells were centrifuged at 1800 rpm for 2 min, flicked and vortexed. 50 ml of surface antibodies diluted in PBS containing 1% FCS (PBS1%) were added and plates incubated for 15–30 min on ice. After washing twice in 160 ml PBS1%, 100 ml of Cytofix/cytoperm (BD Pharmingen) was then added to each well and plates incubated for 10–20 min at 4°C. 100 ml of Perm/Wash (BD Pharmingen), diluted 1∶10 in milliQwater, was added to each well. Plates were washed twice in a further 160 ml of Perm/Wash. 25 ml of intracellular Abs diluted in perm/wash were added to each well and incubated on ice for 15–30 min. Cells were washed twice in Perm/wash and resuspended in 150 ml PBS-1% formalin.

Flow cytometric analysis was performed within 48 h of staining, using a Cyan ADP flow cytometer (BD Biosciences). Data were analyzed using FloJo (version 9). Responses to unstimulated wells were used to establish the gates. Data are presented as % of the total number of cells. This was determined by multiplying the % of IFN-γ^+^ CD4^+^ or CD8^+^ cells of the parent population of CD4^+^ or CD8^+^ cells by the total cell count.

### Statistical Analysis

Data from all immunological assays and BCG challenge experiments were not normally distributed. Consequently, results are presented as medians with interquartile ranges plotted, and non-parametric tests have been applied. Differences in BCG CFU between groups have been analyzed using the Kruskall Wallis test (for comparison of more than two independent groups) and Mann-Whitney U two-sample statistic tests, for comparison of two groups. Paired t-tests have been used for within subject comparisons. Correlations between different parameters were analysed by computing the Spearman's rank correlation test, together with the level of significance. The statistical software used was STATA (Stata Corporation, Texas).

Differences were considered statistically significant, when *P*<0.05. Levels of significance were indicated by asterisk (*P<0.05, ** *P*<0.01 and ***P<0.001). Results are stated in figure legends, unless referred to in the text. All non-parametric data are shown as median±range. Graphs were generated by using Prism 5 software (Version 5.01; GraphPad Software Inc., CA, USA).
